# Advancing Glioblastoma Research with Innovative Brain Organoid-Based Models

**DOI:** 10.3390/cells14040292

**Published:** 2025-02-16

**Authors:** Cátia D. Correia, Sofia M. Calado, Alexandra Matos, Filipa Esteves, Ana Luísa De Sousa-Coelho, Marco A. Campinho, Mónica T. Fernandes

**Affiliations:** 1 Algarve Biomedical Center Research Institute (ABC-RI), Universidade do Algarve, Campus de Gambelas, 8005-139 Faro, Portugal; a57384@ualg.pt (C.D.C.); sofia.am.calado@uac.pt (S.M.C.); macampinho@ualg.pt (M.A.C.); 2 Faculdade de Medicina e Ciências Biomédicas (FMCB), Universidade do Algarve (UAlg), Campus de Gambelas, 8005-139 Faro, Portugal; 3 Faculdade de Ciências e Tecnologia (FCT), Universidade dos Açores (UAc), 9500-321 Ponta Delgada, Portugal; 4 Escola Superior de Saúde (ESS), Universidade do Algarve (UAlg), Campus de Gambelas, 8005-139 Faro, Portugal

**Keywords:** glioblastoma, brain organoids, disease models, drug discovery, human stem cells, tumor heterogeneity, invasion, microenvironment

## Abstract

Glioblastoma (GBM) is a relatively rare but highly aggressive form of brain cancer characterized by rapid growth, invasiveness, and resistance to standard therapies. Despite significant progress in understanding its molecular and cellular mechanisms, GBM remains one of the most challenging cancers to treat due to its high heterogeneity and complex tumor microenvironment. To address these obstacles, researchers have employed a range of models, including in vitro cell cultures and in vivo animal models, but these often fail to replicate the complexity of GBM. As a result, there has been a growing focus on refining these models by incorporating human-origin cells, along with advanced genetic techniques and stem cell-based bioengineering approaches. In this context, a variety of GBM models based on brain organoids were developed and confirmed to be clinically relevant and are contributing to the advancement of GBM research at the preclinical level. This review explores the preparation and use of brain organoid-based models to deepen our understanding of GBM biology and to explore novel therapeutic approaches. These innovative models hold significant promise for improving our ability to study this deadly cancer and for advancing the development of more effective treatments.

## 1. Enduring Challenges in Glioblastoma Research and Therapy

In adult and elderly patients, glioblastoma (GBM) is the most common and aggressive primary malignant brain tumor [1,2,3]. It is classified among gliomas, cancers originating from glial cells or their precursors in the nervous system, specifically as a grade 4 astrocytoma with wild-type isocitrate dehydrogenase 1 or 2 (*IDH1/2*) alleles. These are usually associated with other features, such as the combined gain of chromosome 7 and loss of chromosome 10 (+7/−10), telomerase reverse transcriptase (*TERT*) promoter mutations, epidermal growth factor receptor (*EGFR*) gene amplification, necrosis, and microvascular proliferation [4].

GBM is considered one of the most challenging cancers to treat. Its standard treatment, which includes maximal safe surgical resection combined with radiotherapy and concomitant and adjuvant temozolomide (TMZ) chemotherapy, provides only modest survival benefits [5,6]. Indeed, despite substantial research efforts and the development of new therapies, therapeutic advancements for GBM have remained stagnant over the past two decades [6,7,8,9,10]. Moreover, the prognosis remains poor, characterized by frequent and rapid relapses occurring 6 to 12 months after initial treatment completion [6,11]. The median survival time for patients is typically less than 12 months and the five-year survival rate is around 7% [2,3]. These poor therapeutic outcomes contrast with the significant investment in research and the accumulated knowledge about GBM and are generally attributed to the presence of the blood–brain barrier (BBB), the highly invasive behavior, and the prominent heterogeneity presented by these tumors. Residual cells that remain after treatment often resist TMZ and radiotherapy, further contributing to recurrence and poor therapeutic outcomes [12,13].

Regarding heterogeneity, GBM encompasses clinically relevant subtypes, which warrant personalized approaches to treatment [14,15]. Indeed, each tumor is intrinsically heterogeneous at the genetic, epigenetic, and phenotypic levels. Evidence suggests that the switch between the distinct phenotypic states drives the plasticity of GBM cells and is likely induced by epigenetic changes promoted by therapeutic interventions, including radiotherapy, chemotherapy, and the tumor microenvironment (TME) [12,13]. These factors not only influence the tumor’s adaptability but also contribute to the differentiation of glioblastoma stem-like cells (GSCs) into various tumor-supporting cell types commonly found within the TME, such as pericytes and endothelial cells [16,17,18]. GBM plasticity also may facilitate functional interactions with other cells, including neurons, which can further influence tumor progression and resistance to treatment [19,20,21].

Conventional in vitro models often rely on monolayer cell cultures, which consist of relatively homogeneous cell populations, presenting significant limitations as models for GBM. Tumor spheroids derived from established cell lines or primary cells partially represent GBM complexity and heterogeneity, but lack the three-dimensional structures and interactions with the surrounding microenvironment [22]. Similarly, murine models fail to adequately capture human tumors’ molecular pathology and heterogeneity, mainly related to the human genetic background and brain-specific cell populations [23,24]. These challenges underscore the need for developing, optimizing, and employing more clinically relevant models to investigate GBM mechanisms, identify biomarkers for personalized medicine, and evaluate the effects of new drugs and innovative therapies in preclinical models.

In this context, human stem cells have emerged as a promising tool for developing advanced and more clinically relevant models of GBM. Brain organoid-based models may provide a more accurate representation of these features, making them valuable tools for studying GBM [25]. However, the diversity of available organoid-based models has grown considerably, highlighting the need for a comprehensive comparison to guide researchers in selecting the most appropriate models for their specific applications.

In this review, we provide an in-depth analysis of the current brain organoid-based models derived from human stem cells for studying GBM, focusing on their development and applications, and their impact on advancing research and the understanding of this aggressive brain cancer.

## 2. Human Stem Cells Used in GBM Models

Different types of human stem cells have been used to develop GBM models, from pluripotent stem cells (PSCs), including embryonic stem cells (ESCs) and induced pluripotent stem cells (iPSCs), to multipotent adult stem cells such as neural stem cells (NSCs), with a more restrictive differentiation potential.

### 2.1. Human Embryonic Stem Cells (hESCs) and Induced Pluripotent Stem Cells (hiPSCs)

Human ESCs and iPSCs are considered transformative research tools due to their ability to self-renew and differentiate into any cell type in an adult organism. While hESCs are derived from the inner cell mass of human embryos, hiPSCs are created by reprogramming adult somatic cells like peripheral blood mononuclear cells (PBMCs) or fibroblasts into a pluripotent state using specific transcription factors [26]. Since hiPSCs are obtained without the use of embryonic tissues, these cells offer a more ethically viable alternative to hESCs while retaining similar pluripotent properties. Circumventing the ethical issues of destroying human embryos to obtain hESCs, some hESC lines like H1, H9, and HUES8, were validated, fully characterized, and are commercially available [27]. Fully characterized hiPSC lines are also commercially available from diverse sources, offering standardized options for research and reducing the time and variability associated with generating hiPSCs.

Human ESCs and iPSCs have become invaluable tools for developing GBM models due to their unique properties and versatility. Those cells provide a reliable and consistent source of cells for research that is amenable to genetic modification [28,29,30]. Importantly, iPSCs allow for developing patient-specific GBM models that capture the individual genetic background. Moreover, both hESCs and hiPSCs can be differentiated into neural lineages such as astrocytes, neurons, and neural stem cells, mimicking the TME, and are instrumental in generating brain organoids that replicate the complex three-dimensional architecture of the human brain [29,30,31,32].

### 2.2. Human Expanded Potential Stem Cells (hEPSCs)

A recent study reported the use of human expanded potential stem cells (hEPSCs) [33], a type of stem cell capable of differentiating into both embryonic and extra-embryonic cell lineages. Therefore, these cells have the potential to differentiate not only in the same cell types as hESCs and hiPSCs, but also in cells that contribute to the placenta and other pregnancy-supporting tissues. EPSCs may be derived from pre-implantation embryos or by reprogramming somatic cells using specific culture conditions that enable them to retain a more naive state than conventional PSCs [33,34,35].

Recent studies have reported the application of hEPSCs in GBM research. By differentiating patient-matched normal NSCs and comparing the epigenetic and transcriptional profiles with those of GBM cells, one study provided valuable insights into tumor biology and potential therapeutic targets [36]. Moreover, hEPSCs were used to prepare GBM in vitro models to uncover disease-specific mechanisms and predict drug responses [37]. Still, a clear advantage in using these cells over hESCs or hiPSCs has not yet been demonstrated.

### 2.3. Human Neural Stem Cells (hNSCs)

Human NSCs are multipotent adult cells with a more restricted differentiation potential. They can differentiate into various neural cell types, including neurons and glial cells such as astrocytes and oligodendrocytes [38]. In their native milieu, hNSCs are vital for brain development and neurogenesis.

An advantage of using hNSCs instead of hPSCs (either hESCs or hiPSCs) is that hNSCs are already committed to the neural lineage, simplifying the process of directing their differentiation. Therefore, it eases mimicking brain tumors and the brain microenvironment. Moreover, hNSCs can be genetically modified to introduce mutations commonly associated with GBM, thereby transforming these cells, which supports the hypothesis that NSCs may serve as the cell of origin for GBM [39,40]. Consequently, hNSCs are often used to create in vitro models of GBM, enabling the study of tumor biology and genetic mutations, as well as to test therapeutic approaches [40,41].

NSCs can be sourced from both fetal and adult brain tissues [41,42]. However, their limited supply, challenging accessibility, and ethical considerations often lead to the derivation of early neural progenitor cells (NPCs) from hESCs, hiPSCs, or hEPSCs [36,40,43]. NSCs have been reported to exhibit a more limited proliferative capacity compared to PSCs, which may restrict their use in long-term studies. Additionally, NSC cultures often contain a heterogeneous population of cells at various stages of differentiation, which can further complicate their maintenance and experimental consistency over time [44,45].

In summary, each type of stem cell has its unique advantages and limitations, and the choice of stem cell type depends on the specific research goals. In the context of GBM, the use of hESCs is being progressively replaced by hiPSCs, which provide a more personalized approach by enabling the development of patient-specific models.

## 3. Stem Cell-Derived Brain Organoid-Based Models in GBM Research

Usually, the goal of using stem cells in GBM research is to differentiate them into brain organoids. These are complex structures composed of multiple cell types of the brain, organized into tissues and structures reminiscent of those found in the brain during development, maintained in vitro. Since GBM consists of cancer cells growing in and invading the brain, the stem cell-derived brain organoid-based models are intended to represent the tumor and the organ where it develops, showing more or less representation of the organ, depending on the specific model. Due to some confusion in terminology, it is essential to clarify that these models differ from the so-called tumor organoids, which are created by culturing GBM explants directly from a patient-derived tumor. Tumor organoids closely resemble certain aspects of the original TME. Since they preserve the entire tumor tissue, they comprise intratumoral cells. So, as the malignant cells may be immortalized, the other components of the TME are often lost over time due to subculture. Additionally, these models do not fully replicate the interaction between cancer cells and the surrounding normal brain tissue, as they do not incorporate a co-culture system with normal brain cells.

The human PSCs that are frequently used in these models include hiPSCs reprogrammed in-house or acquired from commercial vendors (e.g., BJiPSC-SV4F-9 cell line, MUNIi008-A, MUNIi009-A, MUNIi010-A, and IMR90), and also hESC lines, usually from commercial vendors (e.g., H1, H9, and HS420) [29,30,32,46,47,48].

**Figure 1:**
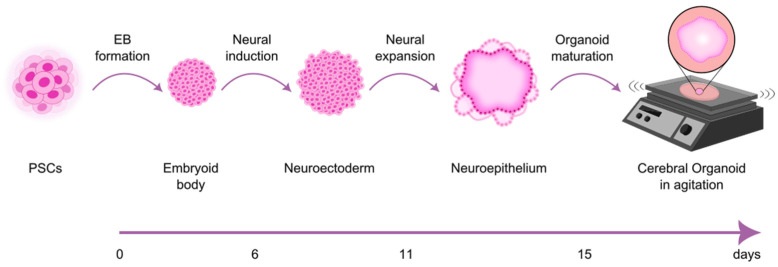
Schematic representation of cerebral organoid generation from human pluripotent stem cells (hPSCs). At day 0, hPSCs are plated in ultra-low attachment plates to form embryoid bodies (EBs). After six days, the EBs are transferred to low-adhesion 24-well plates to induce neural differentiation. Following five days, the developing neuroepithelial tissues are embedded in Matrigel droplets, promoting the expansion of neuroepithelial buds. After an initial stationary growth phase, the organoids are transferred to an orbital shaker or spinning bioreactor to support further maturation. This figure was created based on the Lancaster and Knoblich protocol [50].

There are many different methods to generate brain organoids from stem cells and protocols with several adaptations. These generally replicate the natural developmental processes and involve the use of growth factors or nutrient combinations to induce the formation of organ precursor tissues. Specifically, the generation of cerebral organoids (COs) from PSCs typically follows a specific sequence of steps that begin with standard PSC culture followed by embryoid body (EB) formation, a three-dimensional aggregate that spontaneously differentiates into multiple cell types from the three germ layers, followed by neural induction and neural patterning. The obtained tissue is induced to self-organize by adding extracellular matrix gels, such as Matrigel^TM^, to mimic the three-dimensional (3D) organization of the various cell types like in a tumor or organ [49,50,51], and the organoid is allowed to mature under agitation (Figure 1).

**Figure 2:**
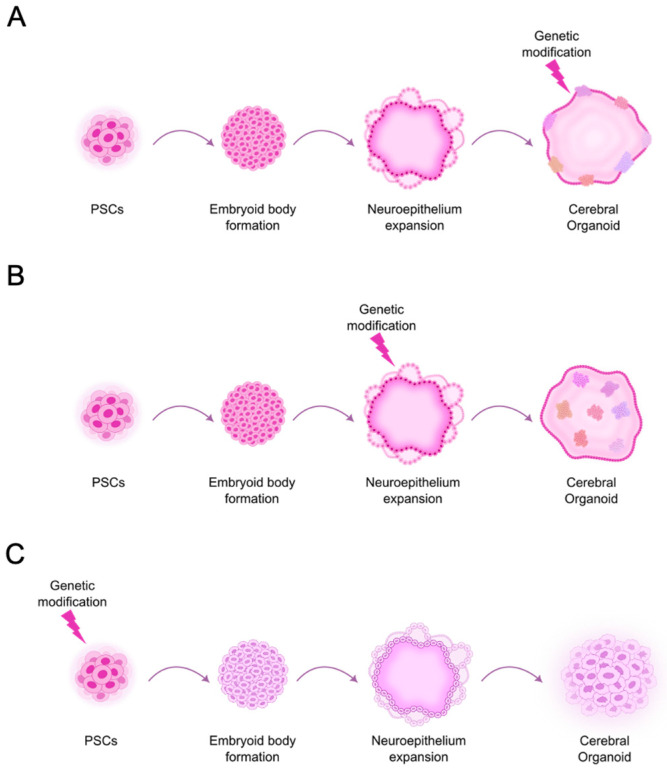
Cerebral organoid (CO)-based GBM models generated through the genetic engineering of human pluripotent stem cells (hPSCs). These models can be developed in three general ways, depending on the stage of CO differentiation at which the genetic modification is introduced. (**A**) When a genetic modification is applied at a later stage, during CO maturation, the resulting model consists of brain-like tissue with focal cancerous growths that originate at the surface of the CO and progressively invade its interior. (**B**) When a genetic modification is introduced during the expansion of neuroepithelial buds, mainly neural stem cells transform, better recapitulating the cells of origin of GBM. This approach leads to the formation of multiple tumors with a limited set of microenvironmental cells. (**C**) When genetic modification is performed at the hPSC stage, the resulting tumor model exhibits a disorganized structure, consisting primarily of transformed cells with only a few microenvironmental cells at later time points. Circular arrows illustrate the differentiation process.

Two main types of clinically relevant stem cell-derived brain organoid-based GBM models have been developed. One of these models allows for the study of GBM initiation, focusing more on the autonomous processes within cancer cells, such as oncogenic mechanisms, tumor heterogeneity, and responses to therapeutic approaches, without explicitly addressing the influence of surrounding tissues or other cell types. It generally implies genetic engineering of PSCs to introduce driver genetic alterations found in GBM, before or in a specific stage of the CO differentiation (Figure 2). Some of the most studied genetic alterations are the ones that are distinctive characteristics of GBM [52].

**Figure 3:**
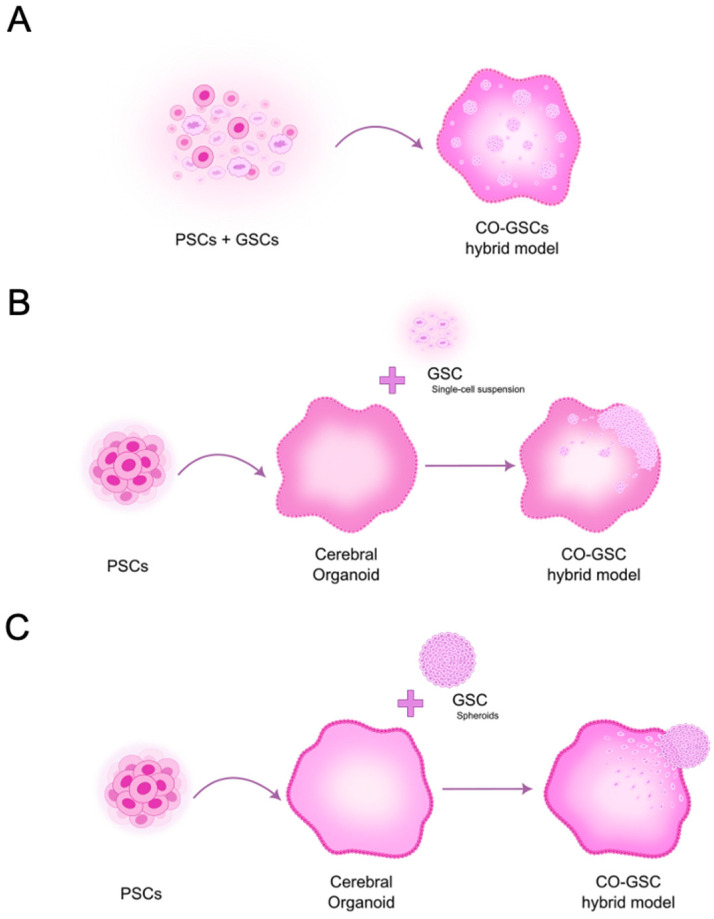
Cerebral organoid (CO)-based GBM models generated through the co-culture of GBM-derived cells with pluripotent stem cells (PSCs) or COs. These models can be developed in three general ways, depending on the timing and method of GBM-derived cell addition. (**A**) Glioblastoma stem-like cells (GSCs) can be mixed with hPSCs before differentiation into COs. (**B**) GSCs can be introduced as a single-cell suspension into preformed COs. (**C**) GSCs can be added as spheroids to COs. Circular arrows represent the differentiation process.

A second model type is generally more suitable for studying advanced GBM, considering its invasion, growth, and interactions with other cellular populations in the brain. It is also used to evaluate the effects of novel therapeutic approaches preclinically. These models may originate from a mixture of glioblastoma stem-like cells (GSCs) and PSCs, which are differentiated into COs (Figure 3A). Alternatively, they may involve co-cultures of GBM cells (either patient-derived primary cells or established GBM cell lines) in single-cell suspensions (Figure 3B) or as spheroids (Figure 3C) tagged by the expression of a fluorescent protein and COs.

The diversity of studies using these two types of models and their applications is described in the following sections.

### 3.1. Models to Dissect GBM Tumor Initiation and Potential Vulnerabilities

GBM usually arises as a primary tumor, being diagnosed when it has already progressed to an advanced stage. As a result, early GBM development cannot be effectively studied using well-established preclinical models derived from these tumors, such as GBM cell lines, tumorspheres, patient-derived cells, or xenograft models. Additionally, in transgenic GBM animal models, detecting and tracking the initial stages of tumor development is challenging [53]. In contrast, in vitro models allow for easier detection and monitoring of tumor initiation and growth, making it more accessible. Therefore, these models are crucial for studying the pathobiology of GBM, developing preventive strategies, improving early diagnostic tools, and identifying potential therapeutic targets.

#### 3.1.1. Development of GBM Models Based on Common Genetic Aberrations to Study Tumor Initiation

The first stem cell-derived organoid-based GBM models enabling the study of tumor initiation recurred to genome-editing techniques like the Clustered Regularly Interspaced Short Palindromic Repeats (CRISPR)/CRISPR-associated (Cas) nuclease 9 (CRISPR/Cas9) technology [54,55] to introduce specific genetic alterations that are commonly found in GBM, affecting genes such as *EGFR*, *NF1*, *TP53*, *TERT*, and *CDKN2A/B* [14,56] (Table 1). The resulting model can differ depending on factors such as how efficiently DNA is transferred into cells, whether the cells are selected and isolated before starting to differentiate, and when the DNA is introduced during differentiation, leading to models with varying proportions of genetically altered cells, including tumor and microenvironmental cells, or even cells from the entire brain where the tumor develops.

One of the first such models was developed by Ogawa and colleagues in 2018 to study tumor initiation and progression [29]. The H9 hESC line (H9-hESCs) was used to prepare COs through the Lancaster and Knoblich protocol [49]. Four months after differentiation, COs were genetically modified using CRISPR/Cas9 to introduce the HRasG12V transgene into the *TP53* gene *locus* via electroporation (Table 1, Figure 2A). This approach aimed to simultaneously express the oncogenic HRasG12V variant while disrupting the *TP53* tumor suppressor *locus* to generate GBM-like tumors, as previously demonstrated in a mouse model [59]. Since the transgene was co-expressed with the tdTomato fluorescent protein reporter, real-time microscopy allowed the observation of tumor development and invasion. The authors found that two months after electroporation, approximately 5% of transformed cells were present on the surface of the COs, which, over the following few months, grew, invaded, and disrupted the CO structure. Furthermore, gene expression analysis indicated that the developed tumors resembled the mesenchymal subtype of human GBM [14,29,56]. The invading cells were further confirmed to be malignant, not only by demonstrating their invasive capabilities to spontaneously fuse and proliferate within normal COs but also by forming tumors when transplanted into the brains of immunodeficient mice. Interestingly, two distinct types of malignancies, previously found in xenograft models, were observed: a highly invasive GBM that fused with and invaded the parenchyma, growing inside normal COs, and a less invasive GBM that grew on the surface of COs. Therefore, COs were proposed as a novel platform for tumor cell transplantation [14,29].

Bian and colleagues created a similar model, termed the neoplastic cerebral organoid (neoCOR), in which tumors were initiated through the introduction of oncogenic mutations using transposon- and CRISPR/Cas9-mediated mutagenesis, along with the inclusion of a GFP reporter [30]. To achieve this, the authors differentiated H9-hESCs into COs. They introduced the plasmids via electroporation at the end of the neural induction phase of the protocol [50], when NSCs and progenitors were expanding on the surface of EBs (Figure 2B). This approach led to specific genetic modification of these cells, which became a minority of transduced cells in 4-month-old COs, in which most analyses were performed. As a result, different neoCORs containing cells with the *CDKN2A*^–^/*CDKN2B*^–^/*EGFR*^OE^/*EGFRvIII*^OE^ genotype, the *PTEN*^–^/*TP53*^–^/*NF1*^–^ genotype, or the *CDKN2A*^–^/*EGFRvIII*^OE^/*PTEN*^–^ genotype, were obtained (Table 1). Compared to normal COs, neoCORs exhibited a significant increase in GFP^+^ cells and were tumorigenic and invasive when transplanted to immunodeficient mice [30]. It is known that tumors are initiated by genetic modification of only a small portion of cells. So, in addition to allowing the study of tumor initiation, neoCORs, composed of a mixed structure containing both tumor and normal tissues, provided an effective model for investigating the interactions between tumor cells and the surrounding normal tissue as well as examining the invasive behavior of tumors [30]. Finally, to determine if the model could be used for targeted drug testing, the authors treated the neoCORs containing cells with the three different genotypes with Afatinib, an EGFR inhibitor. As anticipated, the treatment significantly reduced the number of tumor cells that presented EGFR over-activation in neoCORs [30].

The neoCOR model was later used as a complement to an in vivo orthotopic patient-derived xenograft model for studying the mechanism of action of a specific anticancer therapy [57]. Taubenschmid-Stowers and colleagues used COs engineered to carry mutations of the tumor suppressor genes *TP53*, *NF1*, and *PTEN*, together with GFP labeling, which allowed monitoring of tumor growth over time [30]. They tested a combination of drugs that specifically ablated GFP^+^ tumor cells in the in vitro tumor model, not affecting untransformed cells [57].

These two pioneering models established human stem cell-derived COs as platforms for GBM modeling and study tumor initiation. Moreover, these reports set the basis for developing co-culture models consisting of GBM cells derived from patients and COs to study tumor invasion and the interactions between malignant cells and the brain microenvironment, described in Section 3.2.

Similarly to the previous models, to study the effect of EGFR constitutive activation in the brain, Kim and coworkers introduced the EGFRvIII genetic variant into H9-hESCs using CRISPR/Cas9 before generating COs through the Lancaster and Knoblich protocol [49] (Table 1, Figure 2C). The authors found that it promoted the early differentiation of astrocytes over neurons and enhanced cell proliferation [31]. As a result, COs with constitutively activated EGFR were larger and exhibited a substantial population of astrocytes, whereas the isogenic control did not show any astrocytes even 49 days post-differentiation. In addition, as the EGFRvIII-organoids were sensitive to TMZ treatment, the authors suggested this model could be applied to test potential anti-GBM drugs [31].

Following a slightly different approach, Singh and colleagues developed an inducible model in hESCs (H1 or H9 cell line) by lentiviral transduction of the Tet-On system to express specific short hairpins RNAs (shRNAs) under the promoter of Nestin (*NES*), a gene expressed in NSCs and GSCs, to simultaneously knockdown, *PTEN*, *TP53*, and *NF1* following doxycycline induction [58] (Table 1). These genetically modified hESCs expressing the reporter GFP were differentiated in COs using a modified protocol [46,50,58,60]. Two-month-old COs were treated with doxycycline which led to the formation of cancer-like COs, disrupting the entire organoid architecture in 2 weeks, in contrast to control COs. The cancer-like COs were associated with the proneural GBM subtype [14,56], illustrating the value of the developed model in the study of the initiation and progression of GBM [58].

More recently, to create a model representing different GBM genetic profiles, Wang and coworkers developed human GBM-like organoids termed LEGOs (laboratory-engineered glioblastoma-like organoids) [32]. They used hiPSC lines expressing the GFP reporter, introduced specific genetic alterations using CRISPR/Cas9 ((i.e., *PTEN*^−/−^; *TP53*^−/−^ (named PT), *PTEN*^−/−^; *TP53*^−/−^; *CDKN2A*^−/−^; *CDKN2B*^−/−^ (named PTCC), and also *PTEN*^−/−^; *TP53*^−/−^; *NF1*^−/−^ (named PTN)), and differentiated these cells into COs through the Lancaster and Knoblich protocol [50] (Table 1, Figure 2C). Like Singh et al., *TP53*, *PTEN*, and *NF1* co-disruption was represented, in addition to two other combinations, but in this work, the genetic alterations were already present initially in the PSCs that were used (i.e., hiPSCs instead of hESC) and in all the cells that differentiated from those and composed the CO. All genetically engineered COs grew more than control COs. Moreover, after more than 1 month of maturation, the former exhibited cells with nuclear atypia, which were proved to be tumorigenic in vivo, supporting the notion that these cells were already malignant [32]. To understand how the different genetic mutations affect gliomagenesis, the authors recurred to OMICs technologies to characterize LEGOs at 1 month and 4 months post-differentiation. First, as reported before by Kim et al., in the context of the EGFRvIII genetic alteration, wild-type (WT) organoids’ early differentiation was towards the neuronal lineage. In contrast, the PT and PTCC organoids diverged to astrocytic differentiation. Differently, PTN organoids exhibited both limited neuronal differentiation and reduced astrocytic differentiation, suggesting a neural differentiation blockage. Second, LEGOs recapitulated critical features of cellular heterogeneity that characterize human GBM, and genetic mutations influenced cell phenotypes. PTN organoids were closer to the mesenchymal subtype, and the others were more similar to the proneural subtype. Third, the methylome and metabolome of 4-month LEGOs differed significantly from WT organoids and the DNA methylation alterations and metabolome were dynamic during tumor development and conditioned by the genetic mutations present. Finally, all LEGOs presented premature lipidome differences compared to WT organoids, suggesting that lipidome reprogramming may be a key initial event in gliomagenesis and glycerol lipid metabolism may be a hallmark of GBM. Importantly, these LEGO models exhibit potential as a platform for drug discovery and personalized medicine based on the tumor genotype [32].

Two described models, neoCOR and LEGO, showed potential for quantifying tumor size after modifying the system to include firefly luciferase expression in genetically altered cells, which can be used as a readout [30,32]. This strategy has been implemented in other models and has proven effective in enabling the analysis of a large number of organoids after testing therapeutic approaches [46,61].

#### 3.1.2. Impact of Other Molecular Players on GBM Development

Other models were developed to study the potential role of molecular players on GBM initiation. One of these is the mesenchyme homeobox 2 (MEOX2), a transcription factor critical in embryonic development. Since the *MEOX2* gene localizes in chromosome 7 and was shown to be overexpressed in GBM, the authors suggested it may be one of the oncogenes amplified by chromosome 7 gain driving early GBM development [62,63]. To determine the potential role of MEOX2 in GBM initiation, Schonrock et al. differentiated hiPSCs to COs and, at the stage of cortical induction, these were nucleofected and genetically modified to suppress *PTEN* and *TP53* expression by CRISPR/Cas9 and overexpress *MEOX2*, using a PiggyBac transposon system with the mNeonGreen reporter. The authors found that MEOX2 in combination with the additional mutations enhanced cell proliferation and activated extracellular signal-regulated kinase (ERK) signaling. In addition, MEOX2-enhanced tumor growth in COs was confirmed in vivo by intracranial implantation in immunodeficient mice [64].

Another potential player studied was the methyltransferase-like 7B (METTL7B), a thiol S-methyltransferase, which catalyzes the transfer of a methyl group from S-adenosyl-L-methionine to hydrogen sulfide and other thiol-containing compounds. After finding it differentially expressed between GSCs and hEPSC-derived NSCs, Constantinou and colleagues further explored METTL7B’s role in GBM [36]. To this end, the authors engineered two hEPSC lines to overexpress the gene via lentiviral-mediated transduction and differentiated them into COs, which were maintained for up to 70 days. Although tumor growth was not observed, COs overexpressing METTL7B exhibited altered lineage determination compared to control COs, showing compromised neuronal development and enhanced astroglial differentiation [37].

Altogether, the previous models and their applications allowed identifying essential aspects of gliomagenesis, shedding light on players and pathways involved in GBM heterogeneity and potential targets for therapy. In addition, these models enabled the evaluation of the effects of drugs and other therapeutic approaches in GBM with different genetic profiles setting the base for personalized medicine in GBM. Finally, in models where only a subset of cells is genetically modified for transformation, while the cell populations and microarchitecture of the organoid are partially preserved, it becomes possible to study tumor initiation, progression, and invasiveness.

### 3.2. Models to Study Fully Developed GBM Within the Brain Microenvironment

Cancer cells in tumor–brain organoid co-culture settings often invade deeply and proliferate within the host organoids, resulting in a mosaic of “tumor within the organoid”. While these models have shown promise as a platform to study the tumor microenvironment, their usefulness largely depends on to what extent the host organoids mimic the in vivo tissue. For example, depending on the maturation time, brain organoids may lack mature glial cells, and they generally do not contain vascular and immune cells, all of which are essential components of the brain TME [65].

These models involve the transplantation of human cancer cells from tumors growing in vivo or the transfer of GBM cell lines cultured in vitro. Since only a small proportion of these cells can self-renew and generate tumors, referred to as glioblastoma stem-like cells (GSCs), they must be enriched before co-culture experiments. GSCs are typically enriched and maintained in vitro under non-adherent, serum-free conditions using selective media that promote the growth and maintenance of their stem-like properties, similar to NSC culture conditions. To sustain these cells in culture, the medium must be supplemented with components such as B27 and N2 (branded neural culture supplements), EGF, and basic fibroblast growth factor (bFGF), which support their proliferation and self-renewal. These GSCs are typically maintained as spheroids or tumoroids, which can be used directly in in vitro co-cultures or dissociated to create single-cell suspensions. In addition, they can be transplanted into immunodeficient mice to generate tumor xenografts, enabling the study of tumor growth in vivo. In the co-culture system, the goal is for GSCs to differentiate and form tumors that closely resemble and behave like the original tumor from which the cells were isolated.

Such models allow for the focus on the complex interactions between cancer cells and their surrounding environment, emphasizing how these interactions influence cancer progression and treatment responses, as demonstrated in the studies presented below.

#### 3.2.1. Pioneering Studies Using 3D GBM-Neural Tissue Hybrid Models

In 2013, Nayernia et al. developed an in vitro hybrid model to analyze GBM behavior and the interaction between tumor cells and brain tissue at the molecular level [66]. To this end, patient-derived GSC spheroids containing cells previously stably transduced with tdTomato fluorescent protein were co-cultured in an air/liquid interface with engineered neural tissue (ENT) derived from human ESCs [66,67]. GBM cells survived and grew in neural tissue co-cultures, and some cells migrated from the spheroid, infiltrating into the neural tissue and establishing secondary tumor foci. Moreover, transcriptomic analysis identified over 100 genes upregulated explicitly due to the interaction between the tumor and nervous tissue [66]. This was one of the first GBM models showing the clinical relevance of the 3D co-culture approach. Later, the same group adapted the model to identify miRNA regulators of GBM [68,69].

Using a different approach, Plummer and coworkers developed a heterotypic GBM model consisting of GFP^+^ GBM tumor cells and hiPSC-derived brain cell populations (i.e., neurons and astrocytes). To achieve this, a GBM cell line derived from one patient during resection surgery was mixed with normal NPCs previously differentiated from hiPSCs. Then, the mixed cells were kept under differentiation conditions in non-adherence plates with agitation. The authors combined this model with a high-throughput histology (microTMA) platform to test two drugs (TMZ and Doxorubicin). Specifically, the tumor size and the number of GFP, caspase 3, and Ki67 (an apoptosis and cell proliferation biomarker, respectively) positive tumor cells were used as endpoints to predict drug efficacy. The authors concluded that the developed model helped assess the effects of drugs in primary brain tumor cells from patients and normal neuronal cells, and in detecting and preventing potential adverse effects. Another unique feature of such a model was the possibility of testing drugs on cancer cells derived from a specific patient. Therefore, it is a highly relevant pre-clinical model for cancer drug discovery and personalized medicine, being an alternative or a complement to animal in vivo studies [70].

#### 3.2.2. Innovative Studies Using 3D GBM-Brain Organoid Hybrid Models

Following the example of Ogawa et al. [29], several studies used COs as a platform for tumor cell transplantation instead of mixing patient-derived GBM cells with stem cells before differentiation, similar to how it was performed in the previous model. One such study was developed by Linkous and colleagues, in which they established a novel model system termed glioma cerebral organoid (GLICO). This model involved co-culturing a specific number of GFP-labeled patient-derived GSCs with H1 (or H9)-hESC-derived COs with at least 1 month of maturation (Figure 3B). These were cultured for 24 h and then the cells were allowed to grow and invade for 5 to 16 days, depending on the application [46,71]. They followed the Lancaster and Knoblich protocol [49,50], demonstrating that the model could be prepared using hESCs and hiPSCs. The GLICO model effectively mimics the invasive and proliferative properties of human GBM, forming tumors that closely resemble those observed in patients, including an interconnected network of tumor microtubes involved in cell-to-cell communication. Moreover, this model was shown to reproduce interpatient variability since six GSC lines prepared from tumors of different patients showed distinct patterns of invasion and proliferation, resembling the pattern seen in the original patient specimens. Additionally, GLICOs retained key genetic and signaling components of the parental tumors, often lost in 2D culture [46]. To devise a rapid and quantitative method for repeated in vitro imaging and quantitation of tumor mass in real-time, the authors also genetically modified GSCs to express either firefly luciferase or a secreted *Cypridina* luciferase stably. These were detectable in GLICO tumors by measuring bioluminescence in the tumors or the culture medium. These approaches were used to evaluate the effect of treatments with TMZ, Bis-chloroethylnitrosourea (BCNU), and ionizing radiation on GLICO tumors derived from different patients [46]. Therefore, in addition to providing a manipulable ex vivo system, the GLICO model conserves the complexity of tumor-host interactions and the genetic characteristics of patient tumors, thereby enhancing the potential for studying GBM biology, performing high-throughput drug screening and exploring new therapeutic strategies, and contributing to the development of precision medicine approaches.

By also using the GLICO model, Pine and colleagues compared single-cell RNA sequencing (scRNA-seq) data from tumor cells of five patients to address whether this model could capture the inherent heterogeneity of GBM primary tumors. The results revealed that this model effectively captures the primary tumors’ cellular states and plasticity, being superior to GBM spheroids, tumor organoids, and patient-derived xenografts [72]. In the following study, the same group used the GLICO model to study epigenetics, more specifically chromatin accessibility [47]. Using a combination of single-cell Assay for Transposase-Accessible Chromatin sequencing (scATAC-seq) and scRNA-seq, they found that chromatin accessibility recapitulated GBM transcriptional cellular states that underlie GBM plasticity. Moreover, despite confirming differences between five tumors from different patients, they identified a common cellular compartment comprising neural progenitor-like cells and outer radial glia-like cells (oRG-like), representing populations with stemness properties that may be a common potential target for therapy [47].

The previous studies highlight the importance of a realistic microenvironment for recapitulating the intratumoral heterogeneity and cell state plasticity found in human primary GBM to accurately model GBM and the suitability and convenience of using CO-based models in this context.

Interestingly, the oRG-like cell compartment had been previously identified by Bhaduri and colleagues, who also used scRNA-seq in patient-derived GBM as a specific GSC-related state that was particularly invasive [73]. Since oRG cells are a type of fetal cell that supports stem cell niches in the developing human brain but, in normal conditions, is absent in the adult brain, their presence in these tumors suggests that GBM cells may reactivate developmental programs to enhance their invasiveness. Then, oRG cells were isolated from GBM tumors during resection surgery, labeled with GFP, and co-cultured with iPSC-derived COs, previously differentiated using a modified Sasai’s organoid protocol [74]. The tumor cells engrafted, migrated, and grew inside the organoids for 2 weeks and then were subjected to scRNA-seq. Notably, the diversity of GBM cells identified in the bulk tumors was represented in their model [73]. Therefore, the authors suggested using organoids as tumor allografts to study the biology of GSCs further and for drug testing.

Krieger and colleagues also developed an experimental model using human iPSC-derived COs [49] to study the invasion of four different GFP-labeled patient-derived GSC lines [75]. The authors co-cultured the organoids 24 days post-differentiation. Three days later, organoid invasion and microtube formation were apparent, as it was seen in vivo and in other studies using COs [46,76]. Moreover, to study tumor cell interactions with normal brain cells, early-stage organoids 7 days post-differentiation were dissociated, mixed 1:1 with GSCs, and analyzed 3 days later by scRNA-seq. Although the normal cells represented in the early-stage organoid were probably only NPC and immature neurons, they identified transcriptional changes implicated in the invasion process and potential ligand–receptor interactions between GBM and early-organoid cells [75].

Since GBM organoid-based models allow a great diversity of approaches, Goranci-Buzhala and colleagues developed a study to explore and compare three methods to analyze GSC invasion in human COs [77]. GSCs isolated from primary and recurrent tumors from GBM patients were labeled with mCherry or GFP and COs were differentiated from commercially available hiPSCs using an adapted protocol [78]. In the first method, GSCs were co-cultured with hiPSCs before organoid differentiation (Figure 3A). In the second method, GSCs were added as a single-cell suspension to COs (similarly to the preparation of the GLICO model) (Figure 3B). In these two methods, GBM fusion organoids were analyzed between 10 and 30 days post-differentiation, representing an early stage of development. In the third method, COs were allowed to maturate for 20, 40, or 60 days and then co-cultured with GSC spheroids, which integrated the COs (Figure 3C). Since the GSC spheroids took more time to integrate the younger COs, the authors hypothesized that it could be due to insufficient mitogenic factors secreted by neurons. Indeed, supplementing the culture medium with synaptic protein Neuroligin-3 (NLGN3) to 20-day-old organoids accelerated the integration. On the other hand, the use of GI254023X, an ADAM10 inhibitor, prevented GSC integration into the COs [77]. ADAM10 is an enzyme found on post-synaptic neurons or oligodendrocyte precursor cells, where it facilitates the cleavage of NLGN3, releasing active NLGN3 into the TME [79,80]. Therefore, preventing GSC spheroid integration may indicate interference with GBM invasiveness and/or growth.

The choice of stem cells to be used to prepare brain organoids is of the utmost importance. To investigate this, Azzarelli and colleagues tested three iPSC lines, namely BobC, FSPS-13B, and IMR-90 [81], using the Lancaster and Knoblich protocol for creating microfilament-engineered cerebral organoids (enCORs) [82]. IMR-90 proved to be the most reliable cell line, consistently producing high-quality enCORs 42 days post-differentiation. Notably, the authors employed a commercial kit that included defined serum-free cell culture media and followed a straightforward four-stage protocol, adapted from the method previously established by Lancaster and Knoblich [49,50], to enhance the reproducibility of CO generation. Two different patient-derived GSC lines labeled with GFP were co-cultured with enCORs at two different cell densities (i.e., 10,000 GSCs for 1 CO and 50,000 GSCs for 1 CO) during 24h (Figure 3B), then transferred to fresh culture medium, and finally kept under agitation for 7 days until analysis. As observed in previous reports, GSCs colonized the enCORs, invaded, and represented the tumor heterogenicity found in vivo, sustaining the simultaneous presence of stem/progenitor cells and their differentiated progeny [81].

In a subsequent work to optimize the GLICO model to study GBM invasion, Fedorova and colleagues used the established GBM cell line U87 expressing GFP or tdTomato and CO derived from hiPSCs reprogrammed in-house [48]. Individual spheroids from the GBM U87 cell line were prepared and co-cultured with 55-day-old COs (Figure 3C) in an inclined plane for 3 to 4 days until fusing and then under agitation for 30, 60, and 90 days. Unlike other studies, only after 30 days U87 cells were detected migrating in COs, with most cells being detectable around day 60. Since most of the previously described studies were developed using less mature COs, a stage in which ECM products like Matrigel™ or Geltrex™ are still not resorbed, the authors decided to study the impact of the presence of these products. Therefore, they embedded the co-cultures in these matrices and found that it enhanced the migration of U87 cells inside the COs compared to the ECM-free co-culture system [48]. The significantly longer time needed for migrating GBM cells could also be partially attributed to the nature of the U87 cell line. However, no other established cell lines were tested for comparison. In addition, to allow modeling GBM invasion, U87 spheroid co-culture with COs altered the U87 gene expression profile towards proneural and classical GBM subtypes as shown by bulk mRNA-seq [48]. These results were in accordance with Pine and colleagues’ observations using patient-derived GSC lines. They demonstrate that the CO microenvironment can stimulate significant changes in gene expression even in the well-established GBM cell line U87, which is typically cultured in a 2D environment with fetal bovine serum (FBS) in the culture medium and undergoes multiple passages.

Recently, Nicholson and colleagues developed a model system that more closely recapitulates early gliomagenesis or recurrence after surgery, which they named long-term glioma cerebral organoids (ltGLICOs) [83]. The previous models used a high number of GSCs over a short period and tended to show high concentrations of colocalized GSCs, promoting proliferation and survival, partly through autocrine signaling. So, they are more representative of established and later-stage GBM. The ltGLICOs differ significantly since a small number of patient-derived GSCs are cultured with COs during an extended period, favoring the interactions with brain cells and mechanisms involved in early GBM tumorigenesis or tumor relapse after resection surgery. To develop such a model, the authors co-cultured GSC lines with H1-hESCs (seeding ratio  < 1:1000) before differentiating (Figure 3A) using the microfilament-engineered CO protocol by Lancaster and Knoblich [82] and kept them for 1–24 months in culture [83].

The authors observed a prolonged latency period before GSCs began to proliferate, during which normal organoid development remained unaffected. Over time, as the organoids matured, chronic hypoxia and oxidative stress reshaped the tumor microenvironment, facilitating GSC expansion. Single-cell analysis identified astrocytes, regulated by ischemic signaling networks, as critical players in this process, secreting pro-tumorigenic factors such as FGF1 [83]. This study highlighted how age-related cerebral vascular insufficiency, and chronic hypoxia may drive the heightened aggressiveness of gliomas in older patients.

The use of organoids to model GBM also allowed the study of the interaction of GBM with different brain regions. For example, in a recent study, Fan and colleagues prepared H9-hESCs-derived dorsal forebrain organoids (mainly composed of excitatory neurons), and ventral forebrain organoids (mainly composed of inhibitory neurons) [84] using a previously established protocol [85,86]. These organoids were co-cultured with spheroids obtained from a patient-derived GSC line expressing GFP, and they were included in Matrigel^TM^ in 3D-printed molds with microchannels. The authors found that GBM cells preferentially invaded dorsal forebrain organoids and interacted with neurons so that the gene expression profiles of neurons and GBM were modified [84].

Recent findings by Mangena and colleagues expanded on previous efforts to model GBM using COs, demonstrating widespread mRNA and GFP transfer from malignant to nonmalignant cells via extracellular vesicles. They also provided evidence for the existence of crosstalk between GBM cells and nonmalignant cells. Given the limited availability of models that accurately recapitulate intercellular communication in GBM, this approach provides a valuable tool for studying the crosstalk between GBM and normal brain cells, shedding light on mechanisms of microenvironmental reprogramming [87].

More recently, to overcome the limitations related to the preparation of COs, such as their heterogeneity and low batch quantity that lead to poor reproducibility in scientific studies, Ramani and colleagues developed a protocol for preparing the so-called high-quantity (Hi-Q) brain organoids [88]. Briefly, it consisted of differentiating hiPSC directly into neurospheres (without the intermediate step of preparing EBs) in microwells of a custom-designed spherical plate in cyclo-olefin-copolymer with a round bottom. It was designed to allow identical diffusion conditions for all spheroids and not require precoating. Later, the spheroids were transferred to a spinner bioreactor, and adequate differentiation and maintenance media were used in the different phases to obtain COs [88]. This approach resulted in great numbers of COs per batch (almost 400 CO/batch) and high reproducibility in size even across four independent hiPSC lines. Moreover, time-resolved scRNA-seq demonstrated comparable cell diversity between batches and the relative absence of ectopic stress-inducing pathways. In terms of cellular diversity, six major cell types were represented: neurons, radial glia, proliferating radial glia, astrocytes, and inhibitory and excitatory neurons. These cell populations were similar to those in EB-derived 90- to 180-day-old COs, although the proportion of astrocytes was lower. Moreover, the proliferating cell types (i.e., radial glia and proliferating radial glia) were higher in Hi-Q brain organoids, suggesting their maturation status was slightly behind [88]. This improved method is therefore simple, reliable, and reproducible.

Hi-Q brain organoids were used to prepare an adapted co-culture GBM model to study mCherry-labeled GSC invasion [77,89], which allowed the authors to perform medium-throughput drug screening to identify compounds that can perturb GSC invasion. This analysis identified Selumetinib and Fulvestrant as effective inhibitors of GSC invasion that were confirmed to inhibit GBM invasion in mouse xenografts [88].

In synthesis, the presented advanced 3D co-culture systems offer powerful and versatile models for studying GSC invasiveness and the complexity of GSC heterogeneity, identifying potential molecular targets, and testing therapeutic approaches.

The applications of GBM-CO co-culture 3D models that contributed to GBM research, indicating the general application, aspects related to the CO-based model used in each study, the main findings, and whether these findings were reproduced in in vivo models, are shown in Table 2.

## 4. Enabling Technologies for Analyzing Organoid-Based Models of GBM

In addition to these models being more accessible, technological development has also enabled the use of in-depth microscopy in tissue, 3D reconstruction (whole mount analysis), and spatial omics and single-cell techniques, which can be outsourced to specialized companies at increasingly competitive prices.

### 4.1. Microscopy Technologies

In recent decades, several new microscopy technologies have arisen that can tackle the major technical hurdles organoids impose. The first image-based analysis of organoids was carried out in fixed 2D histological sections [101]. Although informative, this approach diminishes the great advantage that organoids bring to understanding the 3D structure of an organ. However, this has some advantages, given that penetration of antibodies, light scattering, and reduced imaging definition are avoided, thus allowing for images with a high degree of resolution. This is advantageous, especially in cases where single-molecule investigations are to be made.

Regarding fluorescence microscopy, the first approaches to image organoids were carried out with widefield microscopy [102]. Although it allowed an understanding of different aspects of the differentiation of the organoids, it did not allow for adequate 3D reconstruction of the structure. This is mostly because in widefield fluorescent microscopy, sample illumination occurs globally, and the microscope cannot exclude light from out-of-focus planes.

Laser scanning confocal microscopy (LSCM) normally allows the option to allow the collection of images only in the plane of focus, thus allowing for robust 3D reconstruction of the structures imaged. However, LSCM delivers slow acquisition, given that each pixel is obtained individually as a result of scanning; it suffers from the effect of thickening tissue since light-scattering increases in the deepest layers of the tissue, leading to a loss of signal and resolution [103]. Together with tissue clearing, these issues can be reduced in fixed samples. However, using LSCM for live imaging is not the most adequate solution, given that it requires long imaging times, which leads to phototoxicity. Phototoxicity issues during live imaging of organoids can be surpassed using spinning disk confocal microscopy (SDCM). Since each pixel of these images is collected simultaneously using high-sensitivity cameras, exposure time is substantially reduced while resolution is preserved [104,105,106,107,108]. This has been extensively used for high-throughput 3D live imaging of organoids [109]. Nonetheless, SDCM still suffers from considerable light scattering of thick samples, which is the major drawback in organoid cultures, especially in the case of large organoids.

Multi-photon microscopy can penetrate deeper into tissues with reduced phototoxicity. However, given that it is based on point-by-point scanning, it allows for slower imaging. Nonetheless, multi-photon microscopy excels in the 4D imaging of organoids [105]. Another problem of multi-photon microscopy associated with slow acquisition times is the relatively small field of view, which, while enabling 4D imaging of a given section of the organoid, only allows part of the organoid to be imaged. A similar problem arises when using super-resolution fluorescent microscopy. Although it allows for the study of individual molecules in organoids, suffers from reduced fields of view and is associated with high phototoxicity due to the high-intensity lasers necessary for this type of analysis [110,111,112].

Recent advancements in light-sheet fluorescent microscopy (LSFM) enable researchers to surpass these obstacles and harness the full potential of 3D in toto organoid imaging both in fixed and live conditions. The great advantage of LSCM lies in its capacity to image large structures rapidly from cellular to tissue scales, with high signal-to-noise ratios, low phototoxicity, and for a long time. This is due to the excitation of the sample only in a selective plane, thus allowing all fluorescent molecules in that plane to be simultaneously excited, and the photons captured by high-sensitivity cameras, thus reducing the non-specific light from other planes and exposure time while maximizing the field of view. This has provided the means for organoid long-term live high-throughput phenotypic characterization [113] and metabolic activity [114]. Advancements in large-sample histological methods where clearing stands out [115], combined with LSFM, have further increased the capacity to imagine fixed organoids with high-resolution and overall organoid structure. This allows for high-resolution imaging from a cell to tissue/organoid scale. Advances in LSFM have seen the development of lattice-light sheet microscopy (LLSM), where very thin light sheets are sculptured and used to illuminate the sample [116]. These provide faster, lower-intensity illumination of the sample, resulting in minimal photobleaching and phototoxicity, thus increasing spatiotemporal imaging time with minimal detrimental physiological effects. Importantly, LLSM also provides the means to carry out single-molecule imaging, thus extending imaging from single-molecules to tissue scale.

Finally, electron microscopy, specifically high-resolution transmission electron microscopy (TEM), has proven essential for confirming the presence of specific cell types and neuroanatomical features within brain organoids, especially when molecular markers are not always cell type-specific. In one study, TEM was used to examine the detailed structures within the brain organoids, revealing that they contain both glial cells and neurons, with notable features such as myelinated axons and dendrodendritic synapses. These dendrodendritic synapses, which occur between the dendrites of two neurons and facilitate bi-directional signaling, are important microcircuits in the brain and are critical to understanding the functional complexity of the organoid model [46].

### 4.2. Omics Technologies

Omics technologies, including genomics, transcriptomics, proteomics, and metabolomics, offer a comprehensive approach to studying molecular profiles, which is crucial for understanding the mechanisms underlying disease development, interactions between cells and drug responses. Together with advanced single-cell omics approaches, organoids provide an experimental platform for understanding individual cell roles in human organ development and disease. Currently, 42,818 gene expression studies on organoids are available in the NCBI GEO database, of which 6365 are from single-cell omics approaches. This vast amount of data is only now starting to be untapped, and comprehensive understanding and analysis tools for their exploration are in development. New initiatives are being launched that allow the exploration of these resources [117], which will be fundamental for unlocking the full potential of organoid models.

Specifically regarding GBM, some studies have already applied these omics technologies to decipher GBM heterogeneity and cell state transitions [47,72,73,74,75].

By integrating microscopy and omics data, scientists can generate a more holistic understanding of stem-cell-derived organoids, ultimately advancing their use in disease modeling, drug screening, and personalized medicine.

## 5. Challenges, Opportunities, and Future Perspectives

### 5.1. Challenges in Finding the Perfect Model to Study GBM

The poor outcomes of GBM therapies contrast with the investment in basic and translational research, clinical trials, and expensive therapeutic approaches. These are generally attributed to at least some of the previously identified main challenges related to GBM infiltrative nature and to its difficult access, both externally due to the presence of the scull and internally due to the existence of the BBB [118,119]. Moreover, these tumors are characterized not only by inter-tumor heterogeneity, which necessitates personalized approaches to treatment, but also intratumor heterogenicity, both (epi) genetic and phenotypic, which further complicates the development of universally effective treatments [14,56]. The development of effective treatment strategies has been hindered by the limited understanding of the complex biology of GBM and the lack of clinically relevant models as no single model fully captures all relevant aspects of human GBM.

The heterogeneity and intrinsically invasive behavior of GBM cannot be modeled in 2D culture (i.e., GBM established cell lines, patient-derived GBM tumor cells, alone or in co-culture with other brain cells), at least in part due to the lack of ECM and the arrangement of cells in 3D structures as it is found in vivo. Although superior and widely used, GSC-enriched spheroids do not reflect GBM cell state transitions that are largely dependent on the TME clues [22].

Also widely used, GBM rodent models, either transgenic or transplantation-based, can mimic the infiltrative nature of GSCs and their interaction and integration in the brain, fully integrated into a whole organism. However, these models lack physiological relevance in several aspects, primarily due to species differences at both the macro neuroanatomical level (e.g., the underdeveloped murine neocortex) and the cellular level (e.g., variations in astrocyte complexity and the propagation speed of calcium transients in astrocytes) [23]. One specific example of difference directly impacting GBM studies is the relatively high TERT expression in somatic cells, long telomere length, and different promoter sequences of mice compared to humans [120]. Mouse models hinder real-time genetic and molecular studies and present several ethical concerns, limiting their usefulness for both mechanistic research and high-throughput drug screening. Nevertheless, the patient-derived xenograft (PDX) model is the most acceptable for studying human GBM. However, it still presents several limitations, including the necessity of using immunodeficient animals to be possible to transplant human-derived cells, the limited availability of donor tissue, and the acquisition of genetic and epigenetic alterations that make the tumors less representative of the original human tumor [24].

### 5.2. Harnessing Stem Cell-Derived Organoid Biotechnology to Model Glioblastoma

PSC-derived brain organoid models have become valuable tools for studying GBM, providing a more precise representation of human brain development and tumor formation. These 3D models replicate key features of human brain tissue, including cellular diversity, regional specialization and organization, and cell-to-cell interactions, all of which have proven to be crucial for studying tumor biology. They have been shown to exhibit clinically relevant traits such as microtubes and tumor heterogeneity [46,75]. Additionally, although most of the developed models include COs, different brain regions can be represented depending on the culture conditions [50,121,122,123]. Therefore, some studies may test whether the pre-patterning of organoids to other brain regions might influence GSC engraftment and behavior [84]. Since these models are cultured ex vivo, they allow experimental manipulation, drug treatment, and precise control over physiological and environmental factors, making them scalable. They may also include a specific GBM genotype or even patient-derived GBM cells, providing a personalized model [29,30,32,71]. As a result of proven relevance and versatility, these models have become valuable tools in translational research, bringing researchers closer to developing personalized medicine treatments [124].

Of note is that variability across different PSC lines can lead to organoid quality and consistency differences. Some hiPSC lines are more capable of generating brain organoids than others [81]. Then, it is essential to use cell lines with proven quality to generate CO, or in the case a patient-specific line has to be used, to consider specific morphological parameters to assess organoid quality before advancing the study. Moreover, maintaining PSC genomic integrity and stability is mandatory. Measures such as using cells at low passages, culturing with specific media, and using specific methods to evaluate the genetic stability of the lines frequently need to be implemented [125,126].

Genetically engineered stem cell-derived COs were used to study GBM initiation with more or less representation of normal brain cells [29,30,58]. CO with GBM cell transplant co-culture models may be used to investigate the impact of molecular players and/or therapeutic approaches on tumor growth, invasion, and cytotoxicity in full-blown GBM. Although these models, especially the latter ones, have shown promise as a platform for studying the TME in the brain, their effectiveness largely depends on how closely the host organoids replicate the in vivo brain tissue. Nevertheless, depending on the maturation process, key components of the brain TME, such as mature oligodendrocytes and functional neurons, are often absent, and astrocytes may be present in limited quantities [65,111].

In most described reports, the authors used young COs (i.e., 12–42 days post-differentiation). Previous studies have shown that COs and human brains present concordant developmental trajectories, with COs reaching a level of maturation equivalent to approximately 24 weeks post-conception after 6 months in culture [46,127]. Therefore, most described studies have used a model resembling a developing embryonic brain rather than an adult-aged brain, which is characteristic of the target population of GBM. Nevertheless, some studies used 5–6 months COs, which exhibited mature neurons, myelinated axons, dendrodendritic synapses, and glial cells, and when cultured with GSCs, these invaded and developed microtubes and diverse populations of GBM cells contributing to the intrinsic heterogeneity of GBM [46].

Maturate a CO to achieve an adult age is not currently feasible. Nevertheless, organoids cultured in microgravity in the International Space Station (ISS) showed accelerated maturation, with higher levels of genes associated with neural maturity and lower levels of proliferation-associated genes compared to the controls [128]. If human brain organoids age more quickly in microgravity, this could help develop a more accurate model for GBM without needing decades of maturation.

Moreover, the vasculature and a functional BBB are absent in brain organoids, which are critical for studying GBM’s invasiveness and drug delivery, as well as the role of the BBB in restricting therapeutic agents. Additionally, immune cells and other essential components of the TME are often lacking in standard organoid cultures. This absence limits the organoid models’ ability to fully recapitulate the complex immune interactions and inflammation processes seen in GBM.

Therefore, despite their promising potential, considerable challenges must be overcome for these models to fulfill their translational applications fully. To overcome some of these challenges and enhance the predictive value of organoid-based models, they should be complemented with PDX mouse models since these can recapitulate the complexity of in vivo tumors. By combining these approaches, researchers can better understand tumor behavior, therapeutic responses, and potential toxicities. Integrating organoid models with PDX in vivo studies offers a unique opportunity to bridge the gap between laboratory findings and clinical applications. While organoid models provide an excellent platform for studying disease mechanisms and testing novel therapies in a controlled, reproducible environment [88], PDX models help validate these findings in a living organism. Together, these complementary models can accelerate the development of new treatments, improve drug screening strategies, and help bring more effective therapies to clinical trials, ultimately enhancing the success of GBM treatments.

### 5.3. Future Perspectives of Stem Cell-Derived Organoids in Glioblastoma Modeling

The future applications of brain organoid-based models in GBM research hold significant promise, particularly in developing genetically tailored GBM models using individual iPSC clones. Although this approach has not yet been fully applied, the ability to generate patient-specific models could provide a highly accurate representation of a patient’s unique genetic background, allowing for a deeper understanding of disease mechanisms and therapeutic responses. This approach could be particularly valuable in studying cancer predisposition syndromes, offering insights into the genetic factors that increase susceptibility to GBM and other cancers. These models would also be invaluable in studying disease progression and testing potential drug responses in a personalized context, allowing for more effective and targeted treatments.

In the future, these models will be leveraged to deepen the study of the crosstalk between GBM cells and the TME. Understanding how GBM cells manipulate the microenvironment to their advantage could reveal new therapeutic targets to disrupt these interactions. Additionally, there is a need for a detailed analysis of how the cytoarchitecture of the brain influences the invasion routes of GBM cells. Research exploring which specific organoid compartments play a role in GBM’s migratory phenotype will be crucial for designing strategies to limit tumor spread and invasiveness

As technology advances, these organoid-based models are expected to become increasingly powerful tools in understanding and treating GBM, paving the way for precision medicine in the fight against this aggressive brain cancer.

## 6. Conclusions

GBM remains one of the most difficult challenges in cancer treatment due to its aggressive nature, inherent heterogeneity, and the complex environment of the brain. The development of advanced models, particularly those utilizing stem cells and brain organoids, has significantly advanced our understanding of GBM biology and therapeutic responses. The two primary model types discussed—one focusing mostly on GBM initiation and the other on advanced tumor characteristics such as invasion and interaction with surrounding cells—provide valuable insights into the disease. These models, combined with cutting-edge technologies in stem cell/organoid biotechnology, microscopy, and omics, enable improved drug discovery and therapeutic testing.

While each model has its strengths and limitations, their integration continues to enrich our knowledge of GBM, particularly in identifying genetic drivers and evaluating potential treatments. Currently, the combination of organoid-based models with xenograft models represents the most effective strategy for advancing GBM research, as insights from both in vitro and in vivo systems are crucial for the development of successful therapies.

The rapid advancements in stem cell culture methods, genetic engineering tools, and commercial availability of resources have made these models increasingly accessible, reproducible, and ethically compliant thereby enhancing their utility in cancer research. However, significant challenges remain in fully translating the knowledge developed using these models to clinical applications and personalized medicine. Despite these obstacles, their ongoing refinement holds immense potential to drive breakthroughs in GBM treatment. We hope this review serves as a catalyst for further exploration and innovation, inspiring researchers to utilize these models to push the boundaries of GBM knowledge and therapy.

## Figures and Tables

**Table 1:** Models for studying GBM initiation and their characteristics.

Model	Genetic Alterations Introduced	Reporter	hPSCs Used	Ref.
Ogawa’s	Insertion of the HRasG12V transgene into the *TP53* locus, leading to *TP53* disruption.	tdTomato	H9-hESCs	[29]
Bian’s neoCOR	*CDKN2A*^–^/*CDKN2B*^–^/*EGFR*^OE^/*EGFRvIII*^OE^ *PTEN*^–^/*TP53*^–^/*NF1*^–^ *CDKN2A*^–^/*EGFRvIII*^OE^/*PTEN*^–^	GFP	H9-hESCs	[30]
Kim’s	Introduction of the EGFRvIII genetic variant	N/A	H9-hESCs	[31]
Wang’s LEGO	*PTEN*^−/−^; *TP53*^−/−^ *PTEN*^−/−^; *TP53*^−/−^; *CDKN2A*^−/−^; *CDKN2B*^−/−^ *PTEN*^−/−^; *TP53*^−/−^; *NF1*^−/−^	GFP	iPSC lines	[32]
Taubenschmid-Stowers neoCOR	*PTEN*^–^/*TP53*^–^/*NF1*^–^	GFP	H9-hESCs	[57]
Singh’s	Expression of specific shRNAs using the Tet-On to knckdown *PTEN*, *TP53*, and *NF1* knockdown	GFP	H1-hESCs H9-hESCs	[58]

NeoCOR, neoplastic cerebral organoid; N/A, not applicable; LEGOs, laboratory-engineered glioblastoma-like organoids.

**Table 2:** Applications of glioblastoma-cerebral organoid co-culture 3D models.

Application	Model	Main Findings ^1^	In Vivo Study	Ref.
Study Mechanisms of Disease				
Ciliogenesis and its impact on GSC invasiveness	10-day old COs + mCherry-tagged patient-derived GSCs (genetically modified NEK2 conditional KD vs. control)	GSCs induced with cilia (NEK2 KD) failed to infiltrate COs.	Brain xenografts in ID mice	[89]
The role of BCAT1 in GBM proliferation	>25 days old COs + GFP-tagged LN229 established GBM cell line (genetically modified BCAT1 conditional KO vs. control)	BCAT1 is crucial for tumor growth.	Syngeneic mouse model (orthotopic implantation of GBM cells)	[90]
**Test therapeutic approaches**				
Study the therapeutic potential of ZIKV in GBM	6-month-old COs + GFP-tagged GSCs from 2 patient-derived xenografts	ZIKV preferentially infected and presented oncolytic activity against GSCs that expressed high α_v_β_5_ integrin levels. No signs of toxicity to non-cancerous brain cells.	Brain xenografts in ID mice	[91]
Test ZIKV oncolytic effect in CNS malignant tumors	>40 days old COs + GFP-tagged LN18 or U343-MG established GBM cell lines	ZIKV infection led to a significant reduction in tumor cell proportion in COs with GBM. No signs of toxicity to non-cancerous brain cells.	N/D	[92]
Target DHFR to eliminate GSCs	30-day-old COs + 4 patient-derived GFP-tagged GSC lines (i.e., 12M, 25M, 50EF, 53M)	DHFR inhibition in GSCs by pretreatment with methotrexate reduced GSC growth and invasion in COs. No signs of toxicity to non-cancerous brain cells.	N/D	[93]
Test novel BET protein inhibitors for GBM treatment	>35 days old Cos ^2^ + GFP-labeled GBM22 patient-derived xenograft GBM cell line	BET inhibitor UM-002 reduced proliferation and invasion in COs. No signs of toxicity to non-cancerous brain cells.	Brain xenografts in ID mice	[94]
Test a dual Aurora and LIM kinase inhibitor for GBM treatment	>35 days old Cos ^2^ + GFP-labeled GBM22 patient-derived xenograft GBM cell line	Dual Aurora and LIM kinase inhibitor F114 reduced the total number of GFP^+^ cells and invasion in COs. Potential toxicity to non-cancerous brain cells.	N/D	[95]
Test Monensin and derivatives for GBM treatment	>51-day-old Cos ^2^ + RFP-tagged U87MG established GBM cell line	Compound 1 and monensin reduced tumor size and the expression of PARP.	N/D	[96]
Test 5-ALA-mediated photodynamic therapy (PDT) in GBM	41-day-old COs + 2 GFP-tagged GSCs (i.e., GIC7 and PG88)	5-ALA/PDT decreased proliferation and increased apoptosis in cancer cells. No signs of toxicity to non-cancerous brain cells.	N/D	[97]
Target autonomous rhythmic activity of GBM cells	>27-day-old Cos ^2^ + GFP-tagged primary human GSCs (i.e., S24 and BG5)	KCa3.1 inhibitors (TRAM-34 and senicapoc) reduced both global Ca^2+^ activity and tumor cell proliferation. No signs of toxicity to non-cancerous brain cells.	Brain xenografts in ID mice	[98]
Test Tumor Treating Fields (TTFields) in GBM	>27-day-old Cos ^2^ + GFP-tagged primary human GSCs (S24)	TTFields alter tumor cell proliferation and infiltration. Significant reduction in the size of younger (86 days) GBM organoids but not older (159 days).	No	[99]
Test a dual treatment with Rho-kinase inhibitor fasudil and soluble FasL in GBM	hESCs mixed with RFP-tagged U87MG cells and differentiated in COs (used after 75 days)	Decrease in the volume occupied by RFP-labeled U87 cells due to increased apoptosis. No signs of toxicity to non-cancerous brain cells.	Subcutaneous xenografts in ID mice	[100]

BCAT1, branched-chain amino acid transaminase 1; BET, bromodomain and extraterminal domain; COs, cerebral organoids; DHFR, dihydrofolate reductase; GBM, glioblastoma; GSCs, glioblastoma stem-like cells; ID, immunodeficient; Kca3.1, calcium-activated potassium channel; KD, knockdown; KO, knockout; NEK2, NIMA Related Kinase 2; N/D, not performed; Ref., reference; ZIKV, zika virus.^1^ Main findings of the study, considering the specific experimental conditions. ^2^ In this specific study, the maturation age of the COs used in co-cultures is not clearly defined; therefore, an inferred estimate is provided.

## Data Availability

There are no data generated for this manuscript.

## Abbreviations

The following abbreviations are used in this manuscript:

2D	Two-dimensional
3D	Three-dimensional
4D	Four-dimensional
ADAM10	A disintegrin and metalloproteinase domain-containing protein 10
BBB	Blood–brain barrier
BCNU	Bis-chloroethylnitrosourea
Cas9	CRISPR-associated nuclease 9
*CDKN2A/B*	Cyclin-dependent kinase inhibitor *2A/B*
CO	Cerebral organoid
CRISPR	Clustered regularly interspaced short palindromic repeats
EB	Embryoid body
ECM	Extracellular matrix
EGFR	Epidermal growth factor receptor
enCOR	Microfilament-engineered cerebral organoid
EPSC	Expanded potential stem cell
ESC	Embryonic stem cell
ERK	Extracellular signal-regulated kinase
GBM	Glioblastoma
GFP	Green fluorescent protein
GLICO	Glioma cerebral organoid
GSC	Glioblastoma stem-like cell
IDH1/2	Isocitrate dehydrogenase 1 and 2 genes
iPSC	Induced pluripotent stem cell
LEGOs	Laboratory-engineered glioblastoma-like organoids
LLSM	Lattice-light sheet microscopy
LSCM	Laser scanning confocal microscopy
LSFM	Light-sheet fluorescent microscopy
ltGLICOs	Long-term glioma cerebral organoids
MEOX2	Mesenchyme homeobox 2
METTL7B	Methyltransferase-like 7B
NCBI GEO	National Center for Biotechnology Information Gene Expression Omnibus
neoCOR	Neoplastic cerebral organoid
NF1	Neurofibromin 1
NLGN3	Neuroligin-3
NPC	Neural progenitor cell
NSC	Neural stem cell
oRG-like	Outer radial glia-like cells
PSC	Pluripotent stem cell
*PTEN*	Phosphatase and tensin homolog deletions
RFP	Red fluorescent protein
scATAC-seq	Single-cell assay for transposase-accessible chromatin sequencing
scRNA-seq	Single cell RNA sequencing
SDCM	Spinning disk confocal microscopy
SGZ	Subgranular zone
shRNAs	Short hairpins RNAs
SVZ	Subventricular zone
TEM	Transmission electron microscopy
TERT	Telomerase reverse transcriptase
TME	Tumor microenvironment
TMZ	Temozolomide
*TP53*	Tumor Protein 53
